# Supplementary material for: The impact of physical activity variety on physical activity participation
Source: PLoS One. 2025 May 27;20(5):e0323195. doi: 10.1371/journal.pone.0323195 (PMC12112371; doi:10.1371/journal.pone.0323195)
Supplement: S1 Table — (DOCX) [file pone.0323195.s001.docx]

**S1 Table. Means and Standard Deviations for Weekly Minutes of Subjectively and Objectively Measured MVPA by Condition.**

| Condition | Baseline | | 4 Weeks | | 8 Weeks | |
| --- | --- | --- | --- | --- | --- | --- |
|  | M | (SD) | M | (SD) | M | (SD) |
| *Subjective* |  |  |  |  |  |  |
| Variety | 80.00 | (92.11) | 116.91 | (55.57) | 133.74 | (65.70) |
| Consistency | 66.50 | (78.63) | 105.83 | (56.94) | 122.65 | (68.81) |
| Total | 73.72 | (85.36) | 112.05 | (55.74) | 129.03 | (66.40) |
|  |  |  |  |  |  |  |
| *Objective* |  |  |  |  |  |  |
| Variety | 258.93 | (123.80) |  |  | 274.95 | (123.98) |
| Consistency | 241.35 | (136.47) |  |  | 233.98 | (139.41) |
| Total | 251.19 | (127.05) |  |  | 252.89 | (128.74) |

*Note:* ^α^ Difference is marginally significant at *p*<0.10; * Difference is significant at *p*<.05; ** Difference is significant at *p*<.01; *** Difference is significant at *p*<.001; Standard deviations are listed in parentheses.
