# Supplementary material for: The impact of physical activity variety on physical activity participation
Source: PLoS One. 2025 May 27;20(5):e0323195. doi: 10.1371/journal.pone.0323195 (PMC12112371; doi:10.1371/journal.pone.0323195)
Supplement: S2 Table — (DOCX) [file pone.0323195.s002.docx]

**S2 Table. Means and Standard Deviations for Average Weekly MVPA of the Intervention by Condition.**

| Condition | Baseline | | Total MVPA | |
| --- | --- | --- | --- | --- |
|  | M | (SD) | M | (SD) |
| Variety | 80.00 | (92.11) | 127.95^α^ | (46.47) |
| Consistency | 66.50 | (78.63) | 100.81 | (46.04) |
| Total | 73.72 | (85.36) | 115.33 | (47.73) |

*Note:* ^α^ Difference is marginally significant at *p*<0.10; * Difference is significant at *p*<.05; ** Difference is significant at *p*<.01; *** Difference is significant at *p*<.001; Standard deviations are listed in parentheses.
