# Supplementary material for: The impact of physical activity variety on physical activity participation
Source: PLoS One. 2025 May 27;20(5):e0323195. doi: 10.1371/journal.pone.0323195 (PMC12112371; doi:10.1371/journal.pone.0323195)
Supplement: S3 Table — (DOCX) [file pone.0323195.s003.docx]

**S3 Table. Means and Standard Deviations for BREQ-2 by Condition.**

| Variable | Condition | Possible Range | Baseline | | 4 Weeks | | 8 Weeks | |
| --- | --- | --- | --- | --- | --- | --- | --- | --- |
|  |  |  | M | (SD) | M | (SD) | M | (SD) |
| Amotivation | | 0-4 |  |  |  |  |  |  |
|  | Variety |  | 0.42 | (0.59) | 0.62 | (0.41) | 0.71 | (0.66) |
|  | Consistency | | 0.23 | (0.40) | 0.72 | (0.55) | 0.65 | (0.43) |
|  | Total |  | 0.33 | (0.51) | 0.66 | (0.47) | 0.68 | (0.57) |
| External |  | 0-4 |  |  |  |  |  |  |
|  | Variety |  | 1.05 | (0.73) | 0.85 | (0.68) | 1.04 | (0.87) |
|  | Consistency | | 0.89 | (0.88) | 0.93 | (0.65) | 0.68 | (0.58) |
|  | Total |  | 0.98 | (0.80) | 0.88 | (0.66) | 0.89 | (0.78) |
| Introjected | | 0-4 |  |  |  |  |  |  |
|  | Variety |  | 1.97 | (0.97) | 2.12 | (0.87) | 2.17 | (0.76) |
|  | Consistency | | 1.63 | (1.31) | 1.72 | (1.07) | 1.98 | (1.08) |
|  | Total |  | 1.81 | (1.14) | 1.94 | (0.97) | 2.09 | (0.90) |
| Identified |  | 0-4 |  |  |  |  |  |  |
|  | Variety |  | 2.74* | (0.62) | 2.76 | (0.76) | 3.17 | (0.55) |
|  | Consistency | | 2.31 | (0.73) | 2.74 | (0.49) | 2.78 | (0.74) |
|  | Total |  | 2.53 | (0.70) | 2.75 | (0.65) | 3.01 | (0.66) |
| Intrinsic |  | 0-4 |  |  |  |  |  |  |
|  | Variety |  | 2.68 | (0.67) | 2.65 | (0.71) | 3.16 | (0.62) |
|  | Consistency | | 2.33 | (0.87) | 2.50 | (0.63) | 2.72 | (0.79) |
|  | Total |  | 2.52 | (0.78) | 2.59 | (0.67) | 2.98 | (0.72) |

*Note:* ^α^ Difference is marginally significant at *p*<0.10; * Difference is significant at *p*<.05; ** Difference is significant at *p*<.01; *** Difference is significant at *p*<.001; BREQ-2=Behavioral Regulation in Exercise Questionnaire; Standard deviations are listed in parentheses.
