# Supplementary material for: The impact of physical activity variety on physical activity participation
Source: PLoS One. 2025 May 27;20(5):e0323195. doi: 10.1371/journal.pone.0323195 (PMC12112371; doi:10.1371/journal.pone.0323195)
Supplement: S4 Table — (DOCX) [file pone.0323195.s004.docx]

**S4 Table. Means and Standard Deviations for MPAM-R by Condition.**

| Variable | Condition | Possible Range | Baseline | | 4 Weeks | | 8 Weeks | |
| --- | --- | --- | --- | --- | --- | --- | --- | --- |
|  |  |  | M | (SD) | M | (SD) | M | (SD) |
| Enjoyment | | 1-7 |  |  |  |  |  |  |
|  | Variety |  | 4.53 | (1.20) | 4.84 | (0.85) | 5.42 | (0.94) |
|  | Consistency | | 4.50 | (1.51) | 4.60 | (1.22) | 5.09 | (1.36) |
|  | Total |  | 4.52 | (1.34) | 4.74 | (1.02) | 5.28 | (1.13) |
| Competence | | 1-7 |  |  |  |  |  |  |
|  | Variety |  | 4.58 | (1.26) | 4.86 | (0.97) | 5.39 | (1.07) |
|  | Consistency | | 4.83 | (1.47) | 4.67 | (1.30) | 5.28 | (1.17) |
|  | Total |  | 4.70 | (1.36) | 4.78 | (1.12) | 5.34 | (1.10) |
| Appearance | | 1-7 |  |  |  |  |  |  |
|  | Variety |  | 5.17 | (1.18) | 5.13 | (1.43) | 5.21 | (1.22) |
|  | Consistency | | 5.23 | (1.27) | 4.56 | (1.48) | 4.76 | (1.48) |
|  | Total |  | 5.20 | (1.21) | 4.88 | (1.47) | 5.02 | (1.34) |
| Fitness |  | 1-7 |  |  |  |  |  |  |
|  | Variety |  | 5.62 | (0.92) | 5.84 | (0.91) | 6.00 | (0.96) |
|  | Consistency | | 5.92 | (1.21) | 5.93 | (0.80) | 6.05 | (0.77) |
|  | Total |  | 5.76 | (1.07) | 5.88 | (0.86) | 6.02 | (0.87) |
| Social |  | 1-7 |  |  |  |  |  |  |
|  | Variety |  | 3.50 | (1.14) | 3.49 | (1.33) | 3.69 | (1.39) |
|  | Consistency | | 3.22 | (1.53) | 3.48 | (1.40) | 3.60 | (1.50) |
|  | Total |  | 3.36 | (1.33) | 3.48 | (1.35) | 3.65 | (1.42) |

*Note:* MPAM-R=Motives for Physical Activity Measure-Revised; Standard deviations are listed in parentheses.
