# Supplementary material for: The impact of physical activity variety on physical activity participation
Source: PLoS One. 2025 May 27;20(5):e0323195. doi: 10.1371/journal.pone.0323195 (PMC12112371; doi:10.1371/journal.pone.0323195)
Supplement: S5 Table — (DOCX) [file pone.0323195.s005.docx]

**S5 Table. Means and Standard Deviations for PACES by Condition.**

| Condition | Possible Range | Baseline | | 4 Weeks | | 8 Weeks | |
| --- | --- | --- | --- | --- | --- | --- | --- |
|  |  | M | (SD) | M | (SD) | M | (SD) |
|  | 18-126 |  |  |  |  |  |  |
| Variety |  | 83.76 | (28.23) | 127.43 | (41.87) | 173.73 | (81.33) |
| Consistency |  | 85.00 | (25.92) | 115.54 | (38.66) | 138.83 | (74.58) |
| Total |  | 84.34 | (26.89) | 122.21 | (40.43) | 158.90 | (79.49) |

*Note:* PACES=Physical Activity Enjoyment Scale; Standard deviations are listed in parentheses.
