# Supplementary material for: The impact of physical activity variety on physical activity participation
Source: PLoS One. 2025 May 27;20(5):e0323195. doi: 10.1371/journal.pone.0323195 (PMC12112371; doi:10.1371/journal.pone.0323195)
Supplement: S6 Table — (DOCX) [file pone.0323195.s006.docx]

**S6 Table. Means and Standard Deviations for BOSS by Condition.**

| Condition | Possible Range | Baseline | | 4 Weeks | | 8 Weeks | |
| --- | --- | --- | --- | --- | --- | --- | --- |
|  |  | M | (SD) | M | (SD) | M | (SD) |
|  | 0-4 |  |  |  |  |  |  |
| Variety |  | 1.32 | (0.81) | 1.02 | (0.84) | 0.76 | (0.64) |
| Consistency | | 1.22 | (0.97) | 1.17 | (0.89) | 1.00 | (0.79) |
| Total |  | 1.27 | (0.88) | 1.08 | (0.85) | 0.86 | (0.71) |

*Note:* BOSS=Bored of Sports Scale; Standard deviations are listed in parentheses.
