# Supplementary material for: The impact of physical activity variety on physical activity participation
Source: PLoS One. 2025 May 27;20(5):e0323195. doi: 10.1371/journal.pone.0323195 (PMC12112371; doi:10.1371/journal.pone.0323195)
Supplement: S7 Table — (DOCX) [file pone.0323195.s007.docx]

**S7 Table. Means and Standard Deviations for PNSE by Condition.**

| Variable | Condition | Possible Range | Baseline | | 4 Weeks | | 8 Weeks | |
| --- | --- | --- | --- | --- | --- | --- | --- | --- |
|  |  |  | M | (SD) | M | (SD) | M | (SD) |
| Competence |  | 1-6 |  |  |  |  |  |  |
|  | Variety |  | 4.26 | (0.79) | 4.39 | (0.72) | 4.94 | (0.81) |
|  | Consistency | | 4.13 | (1.11) | 4.37 | (0.99) | 4.62 | (1.04) |
|  | Total |  | 4.20 | (0.95) | 4.38 | (0.84) | 4.80 | (0.92) |
| Autonomy |  | 1-6 |  |  |  |  |  |  |
|  | Variety |  | 4.66 | (1.08) | 5.14* | (0.78) | 5.32 | (0.82) |
|  | Consistency | | 4.72 | (1.16) | 4.43 | (1.00) | 4.92 | (0.96) |
|  | Total |  | 4.69 | (1.11) | 4.83 | (0.94) | 5.15 | (0.89) |
| Relatedness |  | 1-6 |  |  |  |  |  |  |
|  | Variety |  | 3.64 | (1.14) | 3.93 | (1.11) | 4.02 | (1.14) |
|  | Consistency | | 3.56 | (1.44) | 3.87 | (1.11) | 3.94 | (1.50) |
|  | Total |  | 3.60 | (1.28) | 3.91 | (1.10) | 3.99 | (1.29) |
| PNSE Total |  | 1-6 |  |  |  |  |  |  |
|  | Variety |  | 12.56 | (1.79) | 13.46^α^ | (1.74) | 14.28 | (1.69) |
|  | Consistency | | 12.41 | (2.51) | 12.67 | (2.30) | 13.48 | (2.49) |
|  | Total |  | 12.49 | (2.14) | 13.11 | (2.02) | 13.94 | (2.08) |

*Note:* ^α^ Difference is marginally significant at *p*<0.10; * Difference is significant at *p*<.05; ** Difference is significant at *p*<.01; *** Difference is significant at *p*<.001; PNSE=Psychological Needs Satisfaction in Exercise Scale; Standard deviations are listed in parentheses.
