# Supplementary material for: The impact of physical activity variety on physical activity participation
Source: PLoS One. 2025 May 27;20(5):e0323195. doi: 10.1371/journal.pone.0323195 (PMC12112371; doi:10.1371/journal.pone.0323195)
Supplement: S8 Table — (DOCX) [file pone.0323195.s008.docx]

**S8 Table. Means and Standard Deviations for PVE by Condition.**

| Condition | Possible Range | Baseline | | 4 Weeks | | 8 Weeks | |
| --- | --- | --- | --- | --- | --- | --- | --- |
|  |  | M | (SD) | M | (SD) | M | (SD) |
|  | 1-6 |  |  |  |  |  |  |
| Variety |  | 3.62 | (1.15) | 4.82* | (0.94) | 5.10** | (0.68) |
| Consistency | | 3.29 | (1.23) | 3.82 | (1.31) | 4.05 | (1.46) |
| Total |  | 3.46 | (1.19) | 4.38 | (1.21) | 4.66 | (1.19) |

*Note:* ^α^ Difference is marginally significant at *p*<0.10; * Difference is significant at *p*<.05; ** Difference is significant at *p*<.01; *** Difference is significant at *p*<.001; PVE=Perceived Variety in Exercise Questionnaire; Standard deviations are listed in parentheses.
