# Supplementary material for: The impact of physical activity variety on physical activity participation
Source: PLoS One. 2025 May 27;20(5):e0323195. doi: 10.1371/journal.pone.0323195 (PMC12112371; doi:10.1371/journal.pone.0323195)
Supplement: S9 Table — (DOCX) [file pone.0323195.s009.docx]

**S9 Table. Means and Standard Deviations for EFI by Condition.**

| Variable | Condition | Possible Range | Baseline | | 4 Weeks | | 8 Weeks | |
| --- | --- | --- | --- | --- | --- | --- | --- | --- |
|  |  |  | M | (SD) | M | (SD) | M | (SD) |
| Engagement |  | 0-12 |  |  |  |  |  |  |
|  | Variety |  | 6.65 | (3.10) | 7.39 | (2.78) | 8.30 | (2.40) |
|  | Consistency | | 6.90 | (2.91) | 7.17 | (2.48) | 7.53 | (3.11) |
|  | Total |  | 6.77 | (2.98) | 7.29 | (2.62) | 7.98 | (2.71) |
| Revitalization |  | 0-12 |  |  |  |  |  |  |
|  | Variety |  | 5.04 | (3.60) | 7.39 | (2.61) | 8.26 | (2.82) |
|  | Consistency | | 5.81 | (3.43) | 6.67 | (2.95) | 7.35 | (3.14) |
|  | Total |  | 5.41 | (3.50) | 7.07 | (2.75) | 7.88 | (2.95) |
| Exhaustion |  | 0-12 |  |  |  |  |  |  |
|  | Variety |  | 6.52 | (3.01) | 4.83 | (2.41) | 4.35 | (2.33) |
|  | Consistency | | 5.57 | (3.11) | 5.67 | (3.11) | 5.12 | (2.40) |
|  | Total |  | 6.07 | (3.06) | 5.20 | (2.73) | 4.68 | (2.36) |
| Tranquility |  | 0-12 |  |  |  |  |  |  |
|  | Variety |  | 5.78 | (2.89) | 7.61 | (2.41) | 8.78** | (2.58) |
|  | Consistency | | 6.95 | (2.85) | 7.06 | (3.00) | 6.88 | (3.00) |
|  | Total |  | 6.34 | (2.90) | 7.37 | (2.66) | 7.98 | (2.89) |

*Note:* ^α^ Difference is marginally significant at *p*<0.10; * Difference is significant at *p*<.05; ** Difference is significant at *p*<.01; *** Difference is significant at *p*<.001; EFI=Exercise-Induced Feeling Inventory; Standard deviations are listed in parentheses.
