# Supplementary material for: The impact of physical activity variety on physical activity participation
Source: PLoS One. 2025 May 27;20(5):e0323195. doi: 10.1371/journal.pone.0323195 (PMC12112371; doi:10.1371/journal.pone.0323195)
Supplement: S11 Table — (DOCX) [file pone.0323195.s011.docx]

**S11 Table. Means and Standard Deviations for FS by Condition.**

|  |  |  | 1 Week | | 4 Weeks | | 8 Weeks | |
| --- | --- | --- | --- | --- | --- | --- | --- | --- |
| Variable | Condition | Possible Range | M | (SD) | M | (SD) | M | (SD) |
| *Pre-Workout* |  |  |  |  |  |  |  |  |
| FS-Current |  | -5-5 |  |  |  |  |  |  |
|  | Variety |  | 0.52 | (1.28) | 1.65 | (1.40) | 1.43 | (1.67) |
|  | Consistency | | 1.50 | -2.16 | 1.78 | (2.10) | 1.76 | (1.60) |
|  | Total |  | 0.98 | (1.79) | 1.71 | (1.72) | 1.58 | (1.63) |
| FS-Enjoyment | | 1-7 |  |  |  |  |  |  |
|  | Variety |  | 3.52 | (0.95) | 4.35 | (1.03) | 4.26 | (0.96) |
|  | Consistency | | 3.95 | -0.51 | 4.28 | (1.07) | 4.41 | (1.06) |
|  | Total |  | 3.72 | (0.80) | 4.32 | (1.04) | 4.33 | (1.00) |
| *Mid- Workout* |  |  |  |  |  |  |  |  |
| FS-Current |  | -5-5 |  |  |  |  |  |  |
|  | Variety |  | 1.22 | (1.78) | 1.91 | (1.47) | 2.48 | (1.62) |
|  | Consistency |  | 1.75 | 1.48 | 2.33 | (1.28) | 2.35 | (1.58) |
|  | Total |  | 1.47 | (1.65) | 2.10 | (1.39) | 2.43 | (1.58) |
| FS-Enjoyment |  | 1-7 |  |  |  |  |  |  |
|  | Variety |  | 4.09 | (0.95) | 4.57 | (1.31) | 4.87 | (1.06) |
|  | Consistency |  | 4.10 | 0.91 | 4.28 | (1.18) | 4.65 | (1.27) |
|  | Total |  | 4.09 | (0.92) | 4.44 | (1.25) | 4.78 | (1.14) |
| *Post-Workout* |  |  |  |  |  |  |  |  |
| FS-Current |  | -5-5 |  |  |  |  |  |  |
|  | Variety |  | 2.22 | (1.45) | 3.22 | (1.28) | 3.09 | (1.20) |
|  | Consistency |  | 2.60 | (1.47) | 2.89 | (1.68) | 3.29 | (1.45) |
|  | Total |  | 2.40 | (1.45) | 3.07 | (1.46) | 3.17 | (1.30) |
| FS-Enjoyment |  | 1-7 |  |  |  |  |  |  |
|  | Variety |  | 4.74 | (1.10) | 5.09 | (1.04) | 5.17 | (1.07) |
|  | Consistency |  | 4.70 | (1.03) | 5.00 | (0.84) | 4.94 | (1.25) |
|  | Total |  | 4.72 | (1.05) | 5.05 | (0.95) | 5.07 | (1.14) |

*Note:* FS=Feeling Scale; Standard deviations are listed in parentheses.
