# Supplementary material for: The impact of physical activity variety on physical activity participation
Source: PLoS One. 2025 May 27;20(5):e0323195. doi: 10.1371/journal.pone.0323195 (PMC12112371; doi:10.1371/journal.pone.0323195)
Supplement: S1 File — (DOCX) [file pone.0323195.s012.docx]

7-Day Physical Activity Recall Interview (7-day PAR)

(Sallis et al., 1985)

You will be asked to indicate any physical activity you did each day for the previous 7 days. The physical activity must be at least moderate (feels like a brisk walk) and for 10 continuous minutes.

You will be asked to label your physical activity as moderate, hard, or very hard activity.

- Moderate would be like a brisk walk. It should feel like you are trying to get out of the rain, running late for an appointment, or trying to catch a bus.
- Hard activity feels like a jog.
- Very hard physical activity feels like a run.

You will be able to record more than one session per day. If your session was two different intensities, you can record this as two different sessions at the different intensities.

The first day you will record is **yesterday**. Then the day before yesterday and so on until you have recorded the last seven days of physical activity.

Questions:

How many days during the previous week did you complete a HIIT workout from the provided website?

Did you do any physical activity yesterday that was at least of moderate intensity (like a brisk walk) and was for at least 10 continuous minutes? [If yes, ask the following questions. If no, move to the next day]

For how many minutes?

Which intensity [Present choices of moderate, hard, and very hard]

Did you do any other physical activity sessions yesterday? [If yes, repeat options above. If no, move to the next day and repeat options above].

Behavioral Regulation in Exercise Questionnaire (BREQ-2)

(Markland & Tobin, 2004)

Why do you engage in exercise? We are interested in the reasons underlying peoples’ decisions to engage, or not engage in physical exercise. Using the scale below, please indicate to what extent each of the following items is true for you. Please note that there are no right or wrong answers and no trick questions. We simply want to know how you personally feel about exercise.

|  | Not true |  | Sometimes true |  | Very true |
| --- | --- | --- | --- | --- | --- |
| 1. I exercise because other people say I should | 0 | 1 | 2 | 3 | 4 |
| 2. I feel guilty when I don't exercise | 0 | 1 | 2 | 3 | 4 |
| 3. I value the benefits of exercise | 0 | 1 | 2 | 3 | 4 |
| 4. I exercise because it is fun | 0 | 1 | 2 | 3 | 4 |
| 5. I don't see why I should have to exercise | 0 | 1 | 2 | 3 | 4 |
| 6. I exercise because my friends/family/partner say I should | 0 | 1 | 2 | 3 | 4 |
| 7. I feel ashamed when I miss an exercise session | 0 | 1 | 2 | 3 | 4 |
| 8. It's important to me to exercise regularly | 0 | 1 | 2 | 3 | 4 |
| 9. I can't see why I should bother exercising | 0 | 1 | 2 | 3 | 4 |
| 10. I enjoy my exercise sessions | 0 | 1 | 2 | 3 | 4 |
| 11. I exercise because others will not be pleased with me if I don't | 0 | 1 | 2 | 3 | 4 |
| 12. I don't see the point in exercising | 0 | 1 | 2 | 3 | 4 |
| 13. I feel like a failure when I haven't exercised in awhile | 0 | 1 | 2 | 3 | 4 |
| 14. I think it is important to exercise regularly | 0 | 1 | 2 | 3 | 4 |
| 15. I find exercise a pleasurable activity | 0 | 1 | 2 | 3 | 4 |
| 16. I feel under pressure from friends/family to exercise | 0 | 1 | 2 | 3 | 4 |
| 17. I get restless if I don't exercise regularly | 0 | 1 | 2 | 3 | 4 |
| 18. I get pleasure and satisfaction from participating in exercise | 0 | 1 | 2 | 3 | 4 |
| 19. I think exercising is a waste of time | 0 | 1 | 2 | 3 | 4 |

Motives for Physical Activities Measure – Revised (MPAM-R)

(Ryan et al., 1997)

The following is a list of reasons why people engage in physical activities, sports, and exercise. Keeping in mind your primary physical activity/sport, respond to each question (using the scale given), on the basis of how true that response is for you.

|  | Not at all true |  |  |  |  |  |  | Very true |
| --- | --- | --- | --- | --- | --- | --- | --- | --- |
| Interest/Enjoyment |  |  |  |  |  |  |  |  |
| 1. It is fun. | 0 | 1 | 2 | 3 | 4 | 5 | 6 | 7 |
| 2. I like to do physical activity. | 0 | 1 | 2 | 3 | 4 | 5 | 6 | 7 |
| 3. It makes me happy. | 0 | 1 | 2 | 3 | 4 | 5 | 6 | 7 |
| 4. I think it is interesting. | 0 | 1 | 2 | 3 | 4 | 5 | 6 | 7 |
| 5. I enjoy the activity. | 0 | 1 | 2 | 3 | 4 | 5 | 6 | 7 |
| 6. I find the activity stimulating. | 0 | 1 | 2 | 3 | 4 | 5 | 6 | 7 |
| 7. I like the excitement of participation. | 0 | 1 | 2 | 3 | 4 | 5 | 6 | 7 |
| Competence |  |  |  |  |  |  |  |  |
| 8. I like engaging in PA which challenges me. | 0 | 1 | 2 | 3 | 4 | 5 | 6 | 7 |
| 9. I want to obtain new skills. | 0 | 1 | 2 | 3 | 4 | 5 | 6 | 7 |
| 10. I want to improve existing skills. | 0 | 1 | 2 | 3 | 4 | 5 | 6 | 7 |
| 11. I like the challenge. | 0 | 1 | 2 | 3 | 4 | 5 | 6 | 7 |
| 12. I want to keep up my current skill level. | 0 | 1 | 2 | 3 | 4 | 5 | 6 | 7 |
| 13. I like activities which are physically challenging. | 0 | 1 | 2 | 3 | 4 | 5 | 6 | 7 |
| 14. I want to get better at my activity. | 0 | 1 | 2 | 3 | 4 | 5 | 6 | 7 |
| Appearance |  |  |  |  |  |  |  |  |
| 15. I want to look or maintain weight so I look better. | 0 | 1 | 2 | 3 | 4 | 5 | 6 | 7 |
| 16. I want to define my muscles so I look better. | 0 | 1 | 2 | 3 | 4 | 5 | 6 | 7 |
| 17. I want to improve my appearance. | 0 | 1 | 2 | 3 | 4 | 5 | 6 | 7 |
| 18. I want to be attractive to others. | 0 | 1 | 2 | 3 | 4 | 5 | 6 | 7 |
| 19. I want to improve my body shape. | 0 | 1 | 2 | 3 | 4 | 5 | 6 | 7 |
| 20. I will feel physically unattractive if I don't. | 0 | 1 | 2 | 3 | 4 | 5 | 6 | 7 |
| Fitness |  |  |  |  |  |  |  |  |
| 21. I want to be physically fit. | 0 | 1 | 2 | 3 | 4 | 5 | 6 | 7 |
| 22. I want to have more energy. | 0 | 1 | 2 | 3 | 4 | 5 | 6 | 7 |
| 23. I want to improve my cardiovascular fitness. | 0 | 1 | 2 | 3 | 4 | 5 | 6 | 7 |
| 24. I want to maintain physical strength for healthy life. | 0 | 1 | 2 | 3 | 4 | 5 | 6 | 7 |
| 25. I want to maintain physical health and well-being. | 0 | 1 | 2 | 3 | 4 | 5 | 6 | 7 |
| Social |  |  |  |  |  |  |  |  |
| 26. I want to be with my friends. | 0 | 1 | 2 | 3 | 4 | 5 | 6 | 7 |
| 27. I like to be with others interested in this activity. | 0 | 1 | 2 | 3 | 4 | 5 | 6 | 7 |
| 28. I want to meet new people. | 0 | 1 | 2 | 3 | 4 | 5 | 6 | 7 |
| 29. I have friends who want me to. | 0 | 1 | 2 | 3 | 4 | 5 | 6 | 7 |
| 30. I enjoy spending time with others doing this activity. | 0 | 1 | 2 | 3 | 4 | 5 | 6 | 7 |

The Psychological Need Satisfaction in Exercise Scale

(Wilson et al., 2006)

Instruction: The following statements represent different experiences people have when they exercise. Please answer the following questions by considering how you typically feel while you are exercising.

|  | False | Mostly False | More false than true | More true than false | Mostly true | True |
| --- | --- | --- | --- | --- | --- | --- |
| PNSE-Perceived Competence |  |  |  |  |  |  |
| 1. I feel that I am able to complete exercises that are personally challenging. | 1 | 2 | 3 | 4 | 5 | 6 |
| 2. I feel confident I can do even the most challenging exercises. | 1 | 2 | 3 | 4 | 5 | 6 |
| 3. I feel confident in my ability to perform exercises that personally challenge me. | 1 | 2 | 3 | 4 | 5 | 6 |
| 4. I feel capable of completing exercises that are challenging to me | 1 | 2 | 3 | 4 | 5 | 6 |
| 5. I feel like I am capable of doing even the most challenging exercises. | 1 | 2 | 3 | 4 | 5 | 6 |
| 6. I feel good about the way I am able to complete challenging exercises. | 1 | 2 | 3 | 4 | 5 | 6 |
| PNSE-Perceived Autonomy |  |  |  |  |  |  |
| 7. I feel free to exercise in my own way. | 1 | 2 | 3 | 4 | 5 | 6 |
| 8. I feel free to make my own exercise program decisions. | 1 | 2 | 3 | 4 | 5 | 6 |
| 9. I feel like I am in charge of my exercise program decisions. | 1 | 2 | 3 | 4 | 5 | 6 |
| 10. I feel like I have a say in choosing the exercises that I do. | 1 | 2 | 3 | 4 | 5 | 6 |
| 11. I feel free to choose which exercises I participate in. | 1 | 2 | 3 | 4 | 5 | 6 |
| 12. I feel like I am the one who decides what exercises I do. | 1 | 2 | 3 | 4 | 5 | 6 |
| PNSE-Perceived Relatedness |  |  |  |  |  |  |
| 13. I feel attached to my exercise companions because they accept me for who I am. | 1 | 2 | 3 | 4 | 5 | 6 |
| 14. I feel like I share a common bond with people who are important to me when we exercise together. | 1 | 2 | 3 | 4 | 5 | 6 |
| 15. I feel a sense of camaraderie with my exercise companions because we exercise for the same reasons. | 1 | 2 | 3 | 4 | 5 | 6 |
| 16. I feel close to my exercise companions who appreciate how difficult exercise can be. | 1 | 2 | 3 | 4 | 5 | 6 |
| 17. I feel connected to the people who I interact with while we exercise together. | 1 | 2 | 3 | 4 | 5 | 6 |
| 18. I feel like I get along well with other people who I interact with while we exercise together. | 1 | 2 | 3 | 4 | 5 | 6 |

Physical Activity Enjoyment Scale (PACES)

(Kendzierski & DeCarlo, 1991)

Please rate how you feel at the moment about physical activity. Below is a list of feelings with respect to physical activity. For each feeling, please mark the number that best describes you.

| 1. I enjoy it | 1 | 2 | 3 | 4 | 5 | 6 | 7 | I hate it |
| --- | --- | --- | --- | --- | --- | --- | --- | --- |
| 2. I feel bored | 1 | 2 | 3 | 4 | 5 | 6 | 7 | I feel interested |
| 3. I dislike it | 1 | 2 | 3 | 4 | 5 | 6 | 7 | I like it |
| 4. I find it pleasurable | 1 | 2 | 3 | 4 | 5 | 6 | 7 | I find it unpleasurable |
| 5. I am very absorbed in physical activity | 1 | 2 | 3 | 4 | 5 | 6 | 7 | I am not at all absorbed in physical activity |
| 6. It’s no fun at all | 1 | 2 | 3 | 4 | 5 | 6 | 7 | It's a lot of fun |
| 7. I find it energizing | 1 | 2 | 3 | 4 | 5 | 6 | 7 | I find it tiring |
| 8. It makes me depressed | 1 | 2 | 3 | 4 | 5 | 6 | 7 | It makes me happy |
| 9. It’s very pleasant | 1 | 2 | 3 | 4 | 5 | 6 | 7 | It's very unpleasant |
| 10. I feel good physically while doing it | 1 | 2 | 3 | 4 | 5 | 6 | 7 | I feel bad physically while doing it |
| 11. It’s very invigorating | 1 | 2 | 3 | 4 | 5 | 6 | 7 | It's not at all invigorating |
| 12. I am very frustrated by it | 1 | 2 | 3 | 4 | 5 | 6 | 7 | I am not at all frustrated by it |
| 13. It’s very gratifying | 1 | 2 | 3 | 4 | 5 | 6 | 7 | It’s not at all gratifying |
| 14. It’s very exhilarating | 1 | 2 | 3 | 4 | 5 | 6 | 7 | It's not at all exhilarating |
| 15. It’s not at all stimulating | 1 | 2 | 3 | 4 | 5 | 6 | 7 | It’s very stimulating |
| 16. It gives me a strong sense of accomplishment | 1 | 2 | 3 | 4 | 5 | 6 | 7 | It does not give me a strong sense of accomplishment |
| 17. It’s very refreshing | 1 | 2 | 3 | 4 | 5 | 6 | 7 | It’s not at all refreshing |
| 18. I felt as though I would rather be doing something else | 1 | 2 | 3 | 4 | 5 | 6 | 7 | I felt as though there was nothing else I would rather be doing |

Bored of Sports Scale (BOSS)

(Wolff et al., 2021)

In the next set of questions, we are interested in your thoughts and feelings when you think about exercising. To answer these questions, please envision yourself during a training session (e.g., working out in the gym). Read each statement and indicate how much you agree with it. (Answers are given on a five-point Likert-scale ranging from strongly disagree to strongly agree.)

| Strongly Disagree Strongly Agree | | | | | |
| --- | --- | --- | --- | --- | --- |
| 1. The training session bores me to death. | 0 | 1 | 2 | 3 | 4 |
| 2. Exercising bores me. | 0 | 1 | 2 | 3 | 4 |
| 3. Exercising is dull and monotonous. | 0 | 1 | 2 | 3 | 4 |
| 4. While doing this boring training session, I spend my time thinking of how time stands still. | 0 | 1 | 2 | 3 | 4 |
| 5. The training session is so boring that I find myself daydreaming. | 0 | 1 | 2 | 3 | 4 |
| 6. I find my mind wandering while I exercise. | 0 | 1 | 2 | 3 | 4 |
| 7. Because I’m bored, I have no desire to exercise. | 0 | 1 | 2 | 3 | 4 |
| 8. I would rather put off this boring training session till tomorrow. | 0 | 1 | 2 | 3 | 4 |
| 9. Because I’m bored, I get tired while exercising. | 0 | 1 | 2 | 3 | 4 |
| 10. The training session bores me so much that I feel depleted. | 0 | 1 | 2 | 3 | 4 |
| 11. While exercising I seem to drift off because it’s so boring. | 0 | 1 | 2 | 3 | 4 |

Perceived Variety in Exercise (PVE) Questionnaire

(Sylvester et al., 2014b)

|  | False | Mostly false | More false than true | More true than false | Mostly true | True |
| --- | --- | --- | --- | --- | --- | --- |
| 1. I feel like I engage in a variety of exercises. | 1 | 2 | 3 | 4 | 5 | 6 |
| 2. I feel like I try a range of exercises. | 1 | 2 | 3 | 4 | 5 | 6 |
| 3. I feel like I change the types of exercise that I do. | 1 | 2 | 3 | 4 | 5 | 6 |
| 4. I feel like my exercise program is varied. | 1 | 2 | 3 | 4 | 5 | 6 |
| 5. I feel like I experience variety in my exercise. | 1 | 2 | 3 | 4 | 5 | 6 |

Exercise-Induced Feeling Inventory (EFI)

(Gauvin & Rejeski, 1993)

**Instructions:** Please use the following scale to indicate the extent to which each word describes how you feel at this moment in time. Record your responses by checking the appropriate box next to each word.

|  | Do not feel | Feel slightly | Feel moderately | Feel strongly | Feel very strongly |
| --- | --- | --- | --- | --- | --- |
| 1. Refreshed | 0 | 1 | 2 | 3 | 4 |
| 2. Calm | 0 | 1 | 2 | 3 | 4 |
| 3. Fatigued | 0 | 1 | 2 | 3 | 4 |
| 4. Enthusiastic | 0 | 1 | 2 | 3 | 4 |
| 5. Relaxed | 0 | 1 | 2 | 3 | 4 |
| 6. Energetic | 0 | 1 | 2 | 3 | 4 |
| 7. Happy | 0 | 1 | 2 | 3 | 4 |
| 8. Tired | 0 | 1 | 2 | 3 | 4 |
| 9. Revived | 0 | 1 | 2 | 3 | 4 |
| 10. Peaceful | 0 | 1 | 2 | 3 | 4 |
| 11. Worn out | 0 | 1 | 2 | 3 | 4 |
| 12. Upbeat | 0 | 1 | 2 | 3 | 4 |

Exercise Self-Efficacy Scale (ESES)

(Bandura, 1977)

This scale instructs participants to answer on a 4-point rating scale how confident they are with regard to carrying out regular physical activities and exercise.

| I am confident…. | Not always true | Rarely true | Moderately true | Always true |
| --- | --- | --- | --- | --- |
| 1) that I can overcome barriers and challenges with regard to physical activity and exercise if I try hard enough | 1 | 2 | 3 | 4 |
| 2) that I can find means and ways to be physically active and exercise | 1 | 2 | 3 | 4 |
| 3) that I can accomplish my physical activity and exercise goals that I set | 1 | 2 | 3 | 4 |
| 4) that when I am confronted with a barrier to physical activity or exercise I can find several solutions to overcome this barrier | 1 | 2 | 3 | 4 |
| 5) that I can be physically active or exercise even when I am tired | 1 | 2 | 3 | 4 |
| 6) that I can be physically active or exercise even when I am feeling depressed | 1 | 2 | 3 | 4 |
| 7) that I can be physically active or exercise even without the support of my family or friends | 1 | 2 | 3 | 4 |
| 8) that I can be physically active or exercise without the help of a therapist or trainer | 1 | 2 | 3 | 4 |
| 9) that I can motivate myself to start being physically active or exercising again after I’ve stopped for a while | 1 | 2 | 3 | 4 |
| 10) that I can be physically active or exercise even if I had no access to a gym, exercise, training, or rehabilitation facility | 1 | 2 | 3 | 4 |

Feeling Scale (FS)

(Hardy & Rejeski, 1989)

This assessment is to be filled out during physical activity. Be sure you select one day this week to you fill this out on a phone, tablet, or computer depending on where you are doing your activity.

| **Immediately before workout** | | **Between minute 10 and 15 during vigorous interval (or anytime between 10 to 15 minutes for moderate)** | |
| --- | --- | --- | --- |
| How do you currently feel?   \|  \| -5 \| Very bad \| \| --- \| --- \| --- \| \|  \| -4 \|  \| \|  \| -3 \| Bad \| \|  \| -2 \|  \| \|  \| -1 \| Fairly bad \| \|  \| 0 \| Neutral \| \|  \| +1 \| Fairly good \| \|  \| +2 \|  \| \|  \| +3 \| Good \| \|  \| +4 \|  \| \|  \| +5 \| Very good \| | Use the following scale to indicate how much you are enjoying this exercise session.   \|  \| 1 \| Not at all \| \| --- \| --- \| --- \| \|  \| 2 \| Very little \| \|  \| 3 \| Slightly \| \|  \| 4 \| Moderately \| \|  \| 5 \| Quite a bit \| \|  \| 6 \| Very much \| \|  \| 7 \| Extremely \| | How do you currently feel?   \|  \| -5 \| Very bad \| \| --- \| --- \| --- \| \|  \| -4 \|  \| \|  \| -3 \| Bad \| \|  \| -2 \|  \| \|  \| -1 \| Fairly bad \| \|  \| 0 \| Neutral \| \|  \| +1 \| Fairly good \| \|  \| +2 \|  \| \|  \| +3 \| Good \| \|  \| +4 \|  \| \|  \| +5 \| Very good \| | Use the following scale to indicate how much you are enjoying this exercise session.   \|  \| 1 \| Not at all \| \| --- \| --- \| --- \| \|  \| 2 \| Very little \| \|  \| 3 \| Slightly \| \|  \| 4 \| Moderately \| \|  \| 5 \| Quite a bit \| \|  \| 6 \| Very much \| \|  \| 7 \| Extremely \| |
|  | | | |
| **During minute 10 and 15 during rest interval (or anytime between 10 to 15 minutes for moderate)** | | **Immediately after workout is completed** | |
| How do you currently feel?   \|  \| -5 \| Very bad \| \| --- \| --- \| --- \| \|  \| -4 \|  \| \|  \| -3 \| Bad \| \|  \| -2 \|  \| \|  \| -1 \| Fairly bad \| \|  \| 0 \| Neutral \| \|  \| +1 \| Fairly good \| \|  \| +2 \|  \| \|  \| +3 \| Good \| \|  \| +4 \|  \| \|  \| +5 \| Very good \| | Use the following scale to indicate how much you are enjoying this exercise session.   \|  \| 1 \| Not at all \| \| --- \| --- \| --- \| \|  \| 2 \| Very little \| \|  \| 3 \| Slightly \| \|  \| 4 \| Moderately \| \|  \| 5 \| Quite a bit \| \|  \| 6 \| Very much \| \|  \| 7 \| Extremely \| | How do you currently feel?   \|  \| -5 \| Very bad \| \| --- \| --- \| --- \| \|  \| -4 \|  \| \|  \| -3 \| Bad \| \|  \| -2 \|  \| \|  \| -1 \| Fairly bad \| \|  \| 0 \| Neutral \| \|  \| +1 \| Fairly good \| \|  \| +2 \|  \| \|  \| +3 \| Good \| \|  \| +4 \|  \| \|  \| +5 \| Very good \| | Use the following scale to indicate how much you are enjoying this exercise session.   \|  \| 1 \| Not at all \| \| --- \| --- \| --- \| \|  \| 2 \| Very little \| \|  \| 3 \| Slightly \| \|  \| 4 \| Moderately \| \|  \| 5 \| Quite a bit \| \|  \| 6 \| Very much \| \|  \| 7 \| Extremely \| |

Week of Workout (select one): Week 1 Week 4 Week 8

Customer Satisfaction Questionnaire

Question 1:

On a scale from 1-7 with 1= Very Dissatisfied and 7= Very Satisfied, please rate your experience with the following:

|  | (1)  Very Dissatisfied | (2)  Dissatisfied | (3)  Somewhat Dissatisfied | (4)  Neutral | (5)  Somewhat Satisfied | (6)  Satisfied | (7)  Very Satisfied |
| --- | --- | --- | --- | --- | --- | --- | --- |
| Overall program |  |  |  |  |  |  |  |
| Website |  |  |  |  |  |  |  |
| Physical activity videos |  |  |  |  |  |  |  |
| Physical activity information from the website |  |  |  |  |  |  |  |
| Counseling sessions |  |  |  |  |  |  |  |
| Information from the physical activity counselor |  |  |  |  |  |  |  |
| Activity logs |  |  |  |  |  |  |  |

Question 2:

What specific features of the program do you think were the most helpful?

Question 3:

What specific features of the program did you think were least helpful?

Question 4:

What aspects of the program would you change? How?

Question 5:

What could be done to improve the website?
