# Supplementary material for: The impact of physical activity variety on physical activity participation
Source: PLoS One. 2025 May 27;20(5):e0323195. doi: 10.1371/journal.pone.0323195 (PMC12112371; doi:10.1371/journal.pone.0323195)
Supplement: Attachment — Submitted filename: Reviewer Comments_20241126.docx [file pone.0323195.s014.docx]

**Reviewer Comments**

Comments for the author

This manuscript, entitled 'The Impact of Physical Activity Variety on Physical Activity Participation,' addresses an important and interesting research issue that provides a reference for promoting physical activity among college-aged individuals. However, several descriptions in the manuscript are unclear and require more detail. In addition, there are too many tables. Please combine them or focus on the most important three to four tables, relegating all others to supplemental tables..

1. In the abstract, it is suggested that the first sentence be rewritten for completion: 'Research indicates that variety (i.e., multiple types of activities) may be effective for increasing physical activity due to...?' Please provide a critical reason to complete the sentence."
2. In the abstract, what is the physical activity variety intervention? Please provide more details.
3. In the Methods section of the abstract, please describe the major outcome indicators investigated in the study and provide details about the primary statistical methods used.
4. In the Conclusion section of the abstract, it is highlighted that 'there was some evidence that variety may lead to increased physical activity...' However, you mentioned that the study results indicated 'Participants in the variety condition reported a marginally significant increase in weekly moderate-to-vigorous physical activity (p = .072).' Please revise the conclusion to reflect that this finding is not statistically significant.
5. On page 3, please change the description from 'type II diabetes' to the updated term 'type 2 diabetes'.
6. On page 3, in the second paragraph, you described that 'Researchers examining physical activity (PA) among young adults and college students often utilize Self-Determination Theory (SDT) to guide interventions.' If this statement is based on previous evidence, using the word 'will' might be inappropriate. Additionally, please provide more details about the examples of PA variety in the following sentence: 'Initial research on PA variety (i.e., multiple types of activities)...'.
7. Regarding the PA variety intervention, you mentioned that limited literature was found. However, there are some studies applied interventions of patient-preferred PA types. Please include the following references and discuss more details about using patient-preferred PA types.

Chiang, S. L., Shen, C. L., Chen, L. C., Lo, Y. P., Lin, C. H., & Lin, C. H.* (2020). Effectiveness of a home-based, telehealth exercise training program for patients with cardiometabolic multimorbidity: A randomized controlled trial. Journal of Cardiovascular Nursing, 35 (5), 491–501.

Chiang, S. L., Shen, C. L., Lee, M. S., Lin, C. H., & Lin, C. H.* (2023). Effectiveness of a 12-week tele-exercise training program on cardiorespiratory fitness and heart rate recovery in patients with cardiometabolic multimorbidity: A randomized controlled trial. Worldviews on Evidence-Based Nursing, 20(4), 339-350.: <https://doi:10.1111/wvn.12607>

Lai, C. Y., Lin, C. H., Chao, T. C., Lin, C. H., Chang, C. C., Huang, C. Y., & Chiang, S. L. (2024). Effectiveness of a 12-week tele-exercise training program in patients with long COVID. Annals of Physical and Rehabilitation Medicine, 67(5), 101853. https://doi.org/https://doi.org/10.1016/j.rehab.2024.101853.

8. On page 4, please expand the discussion in the sentence: 'Furthermore, few studies have examined the efficacy of PA interventions focusing on variety.' Additionally, in the following reference, it is also reported that there was a significant increase in PA self-efficacy.

Lai, C. Y., Lin, C. H., Chao, T. C., Lin, C. H., Chang, C. C., Huang, C. Y., & Chiang, S. L. (2024). Effectiveness of a 12-week tele-exercise training program in patients with long COVID. Annals of Physical and Rehabilitation Medicine, 67(5), 101853. https://doi.org/https://doi.org/10.1016/j.rehab.2024.101853.

9. On page 4, you mentioned, 'Home-based PA is an effective method for college students to engage in enjoyable and sufficient PA,' which I agree with. However, in my opinion, most college students engage in home-based PA rather than using activity facilities. Therefore, home-based PA might not be the issue. Alternatively, you could highlight the prevalence of engaging in activity facilities among college students. Please clarify.
10. Please provide the sample size estimation.
11. Please move the 'Measure' section to the paragraph before 'Data Analysis' and after the 'Intervention' section.
12. PA data was collected using the ActiGraph. Please provide more details about the measurement tool, especially its validity and reliability.
13. On page 9, the paragraph states: 'High-intensity interval training (HIIT) workouts were provided to participants in both conditions....' What are the details of the exercise prescription, including frequency, time, and duration?
14. In the Results section, there are too many tables. Please combine them or focus on the most important three to four tables, and relegate all other tables to supplemental materials.
15. In the Discussion section, the major findings should be discussed, especially in comparison to the previous studies mentioned above.
