# Supplementary material for: The impact of physical activity variety on physical activity participation
Source: PLoS One. 2025 May 27;20(5):e0323195. doi: 10.1371/journal.pone.0323195 (PMC12112371; doi:10.1371/journal.pone.0323195)
Supplement: Attachment — Submitted filename: Response to Reviewers.docx [file pone.0323195.s016.docx]

**Academic Editor:**

1. Please ensure that your manuscript meets PLOS ONE's style requirements, including those for file naming.
   a. The title page now matches the required format and all necessary information is provided. Additionally, headers now match the journal’s preferred format. Finally, the figure file has been converted to a TIFF file.
2. Please include your full ethics statement in the ‘Methods’ section of your manuscript file. In your statement, please include the full name of the IRB or ethics committee who approved or waived your study, as well as whether or not you obtained informed written or verbal consent. If consent was waived for your study, please include this information in your statement as well.
   a. Thank you for catching this. A statement has been added at the end of the first paragraph of the methods section (see final paragraph of page 6) to address these concerns.
3. Please include a separate caption for each figure in your manuscript.
   a. Fig. 1 now has a separate caption that includes the title on page 12 of the manuscript.

**Reviewer A**

*Major concerns:*

1. The sample size (n=47) is small, limiting the ability to detect significant between-group differences.
   a. Thank you for raising this. We agree with this limitation. We have listed sample size as one of the limitations of this study and called for future studies to include larger samples in the discussion section (see paragraph 1 on page 19 and paragraph 2 on page 20).
2. Despite promising trends, key outcomes such as moderate-to-vigorous physical activity (MVPA) and enjoyment lack statistically significant differences between conditions.
   a. We agree this is a fair concern. We have listed this as a limitation in the discussion section (see paragraph 1 on page 19).
3. Marginal significance in several findings (e.g., autonomy, self-efficacy) raises questions about the robustness of conclusions.
   a. We recognize this as a limitation of the manuscript. Therefore, this limitation is discussed in the analysis (see paragraph 1 on page 11) and discussion sections (see paragraph 1 on page 19).
4. The sample is skewed toward female, non-Hispanic participants, limiting applicability to broader populations.
   a. Thank you for pointing this out. We have listed this as a limitation and called for future studies to include more diverse samples in the discussion section (see paragraph 1 on page 19 and paragraph 2 on page 20).
5. The variety intervention was restricted to High-Intensity Interval Training (HIIT) videos, which may not appeal to participants with diverse activity preferences. A broader variety (e.g., yoga, cycling) might yield more compelling results.
   a. We also view this as a limitation to this study. Therefore, we discussed this topic in the limitation section of the discussion section (see paragraph 1 on page 19)

*Minor concerns:*

1. Discrepancies between subjective and objective MVPA underscore potential biases in self-reporting, which should be addressed in future studies.
   b. We recognize this as a limitation. Due to this, we have listed this as a limitation and called for future studies to include more diverse samples in the discussion section (see paragraph 1 on page 19 and paragraph 2 on page 20).
2. Measures such as boredom and enjoyment lack significant between-group differences, contrary to prior studies. This may indicate a need for adjustments in intervention design (e.g., changing activities more frequently).
   a. Thank you for raising this point. We have discussed the potential of the situation described here in the discussion section (see paragraph 1 on page 18).

**Reviewer B**

1. In the abstract, it is suggested that the first sentence be rewritten for completion: 'Research indicates that variety (i.e., multiple types of activities) may be effective for increasing physical activity due to...?' Please provide a critical reason to complete the sentence."
   a. Thank you for pointing this out. We have restructured the first sentence to reflect this recommendation. It now states that we believe a variety intervention is effective due to existing research (see abstract).
2. In the abstract, what is the physical activity variety intervention? Please provide more details.
   a. This is a very good point. We have added further explanation to what the variety and consistency groups received for content and instructions (see abstract).
3. In the Methods section of the abstract, please describe the major outcome indicators investigated in the study and provide details about the primary statistical methods used.
   a. Thank you for catching this. The major outcomes are listed in the first sentence of the abstract methods section and the primary statistical methods used are noted in the final sentence of the section (see abstract).
4. In the Conclusion section of the abstract, it is highlighted that 'there was some evidence that variety may lead to increased physical activity...' However, you mentioned that the study results indicated 'Participants in the variety condition reported a marginally significant increase in weekly moderate-to-vigorous physical activity (p = .072).' Please revise the conclusion to reflect that this finding is not statistically significant.
   a. We agree with this point. This part of the conclusion has been removed (see abstract).
5. 5. On page 3, please change the description from 'type II diabetes' to the updated term 'type 2 diabetes'.
   a. Thank you for catching this. This change has been made (see paragraph 1, page 3).
6. On page 3, in the second paragraph, you described that 'Researchers examining physical activity (PA) among young adults and college students often utilize Self-Determination Theory (SDT) to guide interventions.' If this statement is based on previous evidence, using the word 'will' might be inappropriate. Additionally, please provide more details about the examples of PA variety in the following sentence: 'Initial research on PA variety (i.e., multiple types of activities)...'.
   a. Good point. The word “will has been removed. Additionally, an example of a specific intervention has been added to the third paragraph (see paragraph 2 on page 3).
7. Regarding the PA variety intervention, you mentioned that limited literature was found. However, there are some studies applied interventions of patient-preferred PA types. Please include the following references and discuss more details about using patient-preferred PA types.
   Chiang, S. L., Shen, C. L., Chen, L. C., Lo, Y. P., Lin, C. H., & Lin, C. H.* (2020). Effectiveness of a home-based, telehealth exercise training program for patients with cardiometabolic multimorbidity: A randomized controlled trial. Journal of Cardiovascular Nursing, 35 (5), 491–501.
   Chiang, S. L., Shen, C. L., Lee, M. S., Lin, C. H., & Lin, C. H.* (2023). Effectiveness of a 12-week tele-exercise training program on cardiorespiratory fitness and heart rate recovery in patients with cardiometabolic multimorbidity: A randomized controlled trial. Worldviews on Evidence-Based Nursing, 20(4), 339-350.: https://doi:10.1111/wvn.12607
   Lai, C. Y., Lin, C. H., Chao, T. C., Lin, C. H., Chang, C. C., Huang, C. Y., & Chiang, S. L. (2024). Effectiveness of a 12-week tele-exercise training program in patients with long COVID. Annals of Physical and Rehabilitation Medicine, 67(5), 101853. [https://doi.org/https://doi.org/10.1016/j.rehab.2024.101853](https://doi.org/https:/doi.org/10.1016/j.rehab.2024.101853).
   a. Thank you sending these our way. We have elected to include these studies in the discussion section. Although participants in these studies had access to multiple types of physical activity, they were not specifically instructed to complete different exercises/workouts. Therefore, we felt this would contribute to more appropriately to the variety narrative in the discussion (see paragraph 2, page 16).
8. On page 4, please expand the discussion in the sentence: 'Furthermore, few studies have examined the efficacy of PA interventions focusing on variety.' Additionally, in the following reference, it is also reported that there was a significant increase in PA self-efficacy.
   Lai, C. Y., Lin, C. H., Chao, T. C., Lin, C. H., Chang, C. C., Huang, C. Y., & Chiang, S. L. (2024). Effectiveness of a 12-week tele-exercise training program in patients with long COVID. Annals of Physical and Rehabilitation Medicine, 67(5), 101853. [https://doi.org/https://doi.org/10.1016/j.rehab.2024.101853](https://doi.org/https:/doi.org/10.1016/j.rehab.2024.101853).
   a. Thank you for pointing this out. We have elected not to include this study in the introduction. Although participants in this study had access to multiple types of physical activity, they were not specifically instructed to complete different exercises/workouts. Therefore, we do not feel this would contribute to the variety narrative in the introduction.
9. On page 4, you mentioned, 'Home-based PA is an effective method for college students to engage in enjoyable and sufficient PA,' which I agree with. However, in my opinion, most college students engage in home-based PA rather than using activity facilities. Therefore, home-based PA might not be the issue. Alternatively, you could highlight the prevalence of engaging in activity facilities among college students. Please clarify.
   a. This is a very good point and something we thought would be the case as well. However, research suggests that college student view distance to an activity facility as a significant barrier (cited in this paragraph with citation #24). Therefore, we left the section as is.
10. Please provide the sample size estimation.
    a. Thank you for catching this. A description for while 40 participants was chosen is now included in the introduction (see paragraph 2, page 5).
11. Please move the 'Measure' section to the paragraph before 'Data Analysis' and after the 'Intervention' section.
    a. We appreciate this suggestion. This change has been made.
12. PA data was collected using the ActiGraph. Please provide more details about the measurement tool, especially its validity and reliability.
    a. This is a very good idea. We have added more details regrding the ActiGraph and a sentence establishing validity and reliability (see paragraph 2, page 9).
13. On page 9, the paragraph states: 'High-intensity interval training (HIIT) workouts were provided to participants in both conditions....' What are the details of the exercise prescription, including frequency, time, and duration?
    a. The details for the variety intervention, including the details of the exercises are discussed in the variety section of the procedures (see paragraph 1, page 8).
14. In the Results section, there are too many tables. Please combine them or focus on the most important three to four tables, and relegate all other tables to supplemental materials.
    a. Thank you for pointing this out and we share this concern. We have combined the PA tables into one (see top of page 14). The psychosocial tables have been relegated to supplemental materials as requested.
15. In the Discussion section, the major findings should be discussed, especially in comparison to the previous studies mentioned above.
    a. Thank you for pointing this out. We believe this has been addressed through the incorporation of one of the studies you suggested (see paragraph 2, page 16). The citation is number 48.
