# Supplementary material for: The impact of physical activity variety on physical activity participation
Source: PLoS One. 2025 May 27;20(5):e0323195. doi: 10.1371/journal.pone.0323195 (PMC12112371; doi:10.1371/journal.pone.0323195)
Supplement: Attachment — Submitted filename: Response_to_Reviewers_auresp_2.docx [file pone.0323195.s017.docx]

**Journal Requirements**

1. Please review your reference list to ensure that it is complete and correct. If you have cited papers that have been retracted, please include the rationale for doing so in the manuscript text, or remove these references and replace them with relevant current references. Any changes to the reference list should be mentioned in the rebuttal letter that accompanies your revised manuscript. If you need to cite a retracted article, indicate the article’s retracted status in the References list and also include a citation and full reference for the retraction notice.
   a. Thank you for pointing this out. We have corrected a mistake in that citations 47 and 48 were listed in the incorrect order. This mistake has been fixed on the reference page. Additionally, the citation at the bottom of page 3 has been updated to reflect the reference page with the article from Glaros & Janelle cited as article #20.

**Reviewer 1**

1. The abstract mentions “marginally significant” results (e.g., p = 0.072), which may be misleading for readers. Revise this to reflect the exploratory nature of these findings.
   a. We agree with this point and appreciate the note. The methods and discussion portion of the abstract has been modified to reflect the exploratory nature of this study (see abstract).
2. The description of the intervention and major outcomes in the abstract should be more concise while retaining clarity.
   a. We appreciate you pointing this out. We have cut down both the intervention description and the findings portion of the abstract.
3. In discussing PA variety, the authors cite limited research but fail to integrate all relevant studies fully. Include additional literature.
   a. Thank you for pointing this out. We have added more research to the introduction at the bottom of page 3. Additional citations have also been added in the first paragraph of page 4.
4. Provide more details regarding the ActiGraph device’s reliability and thresholds for defining valid wear-time and MVPA.
   a. We appreciate you for raising this concern and agree with the sentiment. We have added further discussion regarding the two day threshold, why it was used, and what could have been done differently in the limitations section (see paragraph 2, page 19).
5. The counseling sessions’ structure is described, but specific motivational strategies (e.g., goal setting, feedback) should be outlined more clearly.
   a. We agree with this note. A table has been added to pages 7 and 8 that details the individual topics of each session.
6. Some tables (e.g., psychosocial variables) are detailed but dense. Consider condensing tables into key findings and relegating supplementary data to appendices.
   a. We appreciate you pointing this out. We have modified the PA table to now be two supplementary tables to make it more concise and provide clarity (see supplemental tables 1 and 2).
7. Expand on the finding that the consistency group also improved in MVPA and motivation. This suggests that weekly counseling sessions might have driven outcomes, not just the variety intervention. Discuss this as a potential confounder.
   a. Thank you for pointing this out. More detail has been added to the first paragraph of page 18.
8. Highlight the implications for practitioners more clearly, focusing on the feasibility of home-based variety interventions and strategies to enhance adherence.
   a. We agree with this sentiment. A section has been added to the final paragraph on page 21 addressing these concerns.
9. While the authors report significant increases in variables such as self-efficacy, autonomy, and tranquility, many findings are described as “marginally significant” (p-values near 0.10). It would be helpful to reframe these results cautiously and avoid overstating conclusions. Additionally, providing effect sizes for all analyses would clarify the magnitude of observed differences.
   a. Thank you for raising this concern. We have tried to be intentional about using the term “marginal” when discussing these findings to indicate that findings be used cautiously whenever possible. Additionally, we have added to the limitations section to clearly state the concerns you have raised (see page 20, paragraph 1).
   b. Additionally, effect sizes have been added to all relevant findings.
10. The authors report adherence rates and satisfaction scores, which are commendable. However, further details on how participants adhered to completing varied workouts (e.g., participant logs or tracking systems) would strengthen the feasibility findings.
    a. We apologize for not having this included earlier. We have now added a description of how activity was tracked at the bottom of page 8 and top of page 9.
