# Supplementary material for: The impact of physical activity variety on physical activity participation
Source: PLoS One. 2025 May 27;20(5):e0323195. doi: 10.1371/journal.pone.0323195 (PMC12112371; doi:10.1371/journal.pone.0323195)
Supplement: Attachment — Submitted filename: Response_to_reviewers_auresp_3.docx [file pone.0323195.s018.docx]

**Reviewer 1**

Abstract:

1. The use of "marginally significant" (p = 0.072) is misleading. Results should be cautiously interpreted.
   a. Thank you for raising this point. In the conclusion section of the abstract, there is now a disclaimer to interpret findings with caution given the use of marginal significance (see abstract).
2. The conclusion should briefly suggest practical applications of the findings.
   a. We appreciate you raising this point. The conclusion of the abstract now concludes with practical applications of the findings related to supporting client psychological needs and providing home-based physical activity options (see abstract).

Introduction

3. Some references are outdated (e.g., pre-2010 studies). Recent research (2022-2024) should be included.
   a. Thank you pointing this out. Study citation #4, 13, and 28 have been updated with more recent sources that provide updated information on the topic. Due to the limited number of studies that examined variety in physical activity program previously, authors deemed studies discussing this topic necessary to keep as they help set up the research gap.
4. The research gap should be more explicitly stated.
   a. We feel this is a great point. We have gone back to the introduction to clarify the gaps this project was addressing and wrote them as a bridge to the purpose of the study (see top of page 5).

Methods

5. Sample size (n = 47) is small, reducing generalizability.
   a. Thank you for raising this point. In the limitations section of the discussion, we have added more on why we used a small sample size. The reason being this study was a feasibility and acceptability study (see page 20, first paragraph).
6. Lack of power analysis to justify sample size.
   a. Following the previous point, we also discussed why the small sample size used in a feasibility and acceptability study meant it would not be appropriate to have a power analysis. This is discussed as a limitation (see page 20, first paragraph).
7. Weekly counseling sessions for both groups could be a confounder—this should be discussed in limitations.
   a. We appreciate this point. We have added a sentence discussing this confounder to the discussion section (see page 20, second paragraph).

Results

8. Many findings are described as "marginally significant" (p-values near 0.10). These should be cautiously framed.
   a. We agree with this point and have addressed this issue in the abstract and the limitations section to clarify why this term was used (see abstract and page 20, first paragraph)
9. Effect sizes should be reported consistently.
   a. Thank you for catching this. We have no added effect sizes for significant findings throughout the document.
10. No post-hoc tests or multiple comparison corrections were mentioned.
    a. We appreciate you raising this concern. However, due to the small sample size and exploratory nature of this study, post-hoc tests were not used to avoid the risk of over misinterpretation.

Discussion

11. Does not critically assess why some hypotheses were not supported.
    a. We agree with this concern. Discussion has been added to various sections of the discussion section where an unsupported hypothesis is discussed (see pages 18-19).
12. The role of PA enjoyment and boredom reduction needs deeper exploration.
    a. Thank you for raising this point. Further clarification has been added to this discussion to talk about how enjoyment and boredom relate to other components and findings in this study (see page 19, paragraph 2).
13. Future research directions should be more specific.
    a. We appreciate this point being brought to our attention. We have clarified some specific strategies in which future research directions could move (see pages 21-22, final paragraph-first paragraph).

Conclusion

14. Practical applications are vague—how can practitioners use these findings?
    a. Thank you for raising this concern. The practical applications have been clarified (see end of page 22).
15. Should clearly discuss policy or intervention implications.
    a. Thank you for raising this. We feel that the clarification of intervention implications in the discussion section has provided clarity to the discussion of this during the conclusion.

References

16. Some references are outdated (pre-2010).
17. A few recent studies (2022-2024) should be included to reflect current trends.
    a. To address both of these points, where suitable, older studies were replaced with more recent ones to reflect the current environment. However, do the limited nature of studies similar to the present one, some integral older studies are still featured.
